# La reutilización de biomarcadores para otros fines: oportunidades y dificultades en el cambiante campo de la medicina de laboratorio

**DOI:** 10.1515/almed-2024-0196

**Published:** 2025-01-03

**Authors:** Damien Gruson, Nassiba Menghoun, Anne-Catherine Pouleur, Antoni Bayes Genis

**Affiliations:** Servicio de Medicina de Laboratorio, Cliniques Universitaires St-Lux, (Bruselas, Bélgica) y Universidad Católica de Louvain, Bruselas, Bélgica; Red de Investigación en Endocrinología, Diabetes y Nutrición, Instituto de Investigación Experimental y Clínica, Cínicas Universitarias Saint-Luc y Universidad Católica de Louvain, Bruselas, Bélgica; Servicio de Medicina Cardiovascular, Cínicas Universitarias Saint-Luc Saint-Luc, Bruselas, Bélgica; Red de Investigación en Enfermedades Cardiovasculares (CARD), Instituto de Investigación Experimental y Clínica (IREC), Universidad Católica de Louvain (UCLouvain), Bruselas, Bélgica; Instituto del Corazón de Germans Trias (iCor), Pujol, Universitat Autònoma de Barcelona, CIBERCV, Barcelona, España

**Keywords:** CA125, biomarcador, reutilización, insuficiencia cardíaca, congestión e inflamación, diagnóstico clínico

Estimado Editor,

Existe un interés creciente en el concepto de reutilización de fármacos, consistente en emplear fármacos ya existentes para nuevos fines terapéuticos, ya que esta estrategia podría facilitar el desarrollo de tratamientos más rápidos y rentables [1], 2]. Una tendencia similar, la reutilización de biomarcadores, está suscitando el interés de la comunidad científica, ya que se trata de un método innovador, a través del cual se emplean biomarcadores ya existentes en aplicaciones diferentes a las establecidas originariamente [3]. Un ejemplo de ello es el biomarcador antígeno de carbohidratos 125 (CA125), tradicionalmente asociado al cáncer de ovario. Este biomarcador está demostrando potencial para el tratamiento de la insuficiencia cardíaca (IC), una patología en la que la utilidad de ciertos biomarcadores ha sido sobradamente demostrada [4], 5]. De hecho, los péptidos natriuréticos, el péptido natriurético de tipo B (BNP) y proBNP N-terminal (Nt-proBNP), forman parte del panel de biomarcadores empleados habitualmente en el diagnóstico de la IC [6]. Esta evolución suscita nuevos interrogantes sobre la forma en la que se podrían reutilizar biomarcadores existentes para mejorar nuestra comprensión de las nuevas patologías, lo que aceleraría el desarrollo de métodos diagnósticos y terapéuticos innovadores.

CA125: Del cáncer de ovario a la insuficiencia cardíaca: El antígeno de carbohidratos 125 (CA125) es un biomarcador cuyo uso para fines diagnósticos y terapéuticos en el cáncer de ovario es sobradamente conocido, siendo así mismo empleado en el control de progresión de la enfermedad y la respuesta a tratamiento [7]. Estudios recientes subrayan la utilidad de este biomarcador en otros contextos clínicos, concretamente, en la insuficiencia cardíaca (IC). Esta glicoproteína, codificada por el gen MUC16 y sintetizada por células mesoteliales, está implicada en procesos como la inmunidad mediada por células, la inflamación y el transporte de fluidos [4], 8]. Cabe señalar que dichos mecanismos se solapan de manera significativa en el cáncer y la IC, lo que indica que ambas patologías comparten vías como la inflamación, la congestión y la fibrosis.

En la IC, los niveles de CA125 se encuentran elevados, especialmente en los pacientes con insuficiencia cardíaca derecha o con congestión significativa, lo que refleja cambios hemodinámicos como la retención de líquidos y el desarrollo de edemas. Estos cambios podrían reflejar la existencia de un mecanismo patofisiológico común al cáncer y la IC, especialmente en las patologías caracterizadas por la activación de células mesoteliales y el desequilibrio hídrico [9]. En el presente estudio, se profundiza en el conocimiento actual sobre el CA125 más allá de su uso habitual, ofreciendo una nueva perspectiva sobre su papel en los procesos de las enfermedades sistémicas.

Así mismo, en los pacientes con IC, CA125 se postula como un valioso marcador para evaluar la gravedad de la enfermedad y ofrecer una orientación para la toma de decisiones terapéuticas. Por ejemplo, se ha establecido una correlación significativa entre los niveles elevados de CA125 y la fibrosis, tal como subraya su asociación con marcadores como el propéptido natriurético cerebral N-terminal (NT-proBNP) y el factor de crecimiento de fibroblastos 23 (FGF-23), especialmente en la IC con fracción de eyección preservada (HFpEF) [8], 10]​. Estos hallazgos subrayan su potencial como herramienta pronóstica en el tratamiento de la IC.

Además del cáncer de ovario, los usos clínicos de CA 125 podrían ampliarse a otras neoplasias, incluyendo el mesotelioma pleural maligno y otros tumores epiteliales. La afectación de las células mesoteliales y la retención de líquidos en el derrame pleural indican una posible aplicación del CA125 como marcador diagnóstico o pronóstico en estas patologías. Su relevancia en diferentes enfermedades sugiere la posibilidad de reutilizar biomarcadores como CA125 en diversos contextos clínicos, permitiendo al clínico tratar patologías complejas como la IC y, posiblemente, otras neoplasias con mayor precisión. Los estudios recientes respaldan el empleo de CA125 como biomarcador en la intersección entre la oncología y la cardiología, ofreciendo una estrategia única a la hora de explorar los mecanismos comunes al cáncer y a las enfermedades cardiovasculares [11].

El caso de la reutilización de biomarcadores: La reutilización de biomarcadores consiste en la identificación y validación de biomarcadores que inicialmente fueron validados para una enfermedad y aplicarlos a otra. Esta estrategia ofrece múltiples ventajas, entre las que destaca una reducción del tiempo y los costes asociados al desarrollo y validación desde cero de nuevos biomarcadores. Tal como ocurre con la reutilización de fármacos, en la reutilización de biomarcadores se aprovecha el conocimiento e infraestructura ya existentes en relación a un biomarcador, acelerando de este modo la transferencia de un descubrimiento científico a la práctica clínica [2]​. En el caso que nos ocupa, la reutilización del CA125 en el campo de la cardiología, cuando se venía empleando únicamente en el área de la oncología, refleja una conciencia creciente entre la comunidad científica de que algunas patologías comparten mecanismos patofisiológicos. Por ejemplo, la inflamación, la remodelación de tejidos y la fibrosis son vías implicadas tanto en el cáncer como en la insuficiencia cardíaca, lo que justificaría los estudios sobre la función del CA125 en las enfermedades cardiovasculares [4], 10]​. Al reconocer estos mecanismos comunes, la comunidad científica podrá aplicar los biomarcadores en diferentes patologías, facilitando así el desarrollo de nuevas herramientas diagnósticas y terapéuticas.

Limitaciones en la reutilización de biomarcadores: Aunque la reutilización de biomarcadores resulta prometedora, no está exenta de algunas dificultades. Un aspecto clave es la necesidad de una validación general en diferentes patologías. Por ejemplo, aunque se ha demostrado la utilidad del CA125 en la IC, su función en este nuevo contexto requiere ser validada, para garantizar que proporciona información clínica relevante en la práctica clínica [4], 8].

Otra dificultad radica en comprender los fundamentos de la mecánica del biomarcador. Los biomarcadores como el CA125 podrían mostrar un comportamiento diferente, dependiendo de la enfermedad, debido a variaciones en los mecanismos patofisiológicos subyacentes. En el cáncer de ovario, los niveles de CA125 están correlacionados con la carga tumoral, mientras que en la IC reflejan principalmente congestión e inflamación sistémica [10]. Dichas diferencias precisan de estudios mecanísticos rigurosos, para garantizar que los biomarcadores reutilizados continúan siendo fiables y precisos. Así mismo, su aplicación clínica requiere de un análisis cauteloso de la forma en la que se va a integrar el biomarcador en los protocolos diagnósticos existentes. En el caso del CA125, su amplia disponibilidad y rentabilidad lo convierten en un candidato atractivo a ser empleado en la IC en la práctica clínica. No obstante, aún resta abordar ciertas cuestiones prácticas relativas a su lugar en los algoritmos de tratamiento, así como en los protocolos de tratamiento del paciente [4]. Aunque la mayoría de las pruebas de medición de CA125 ostentan la etiqueta CE, el reglamento sobre diagnóstico *in vitro* (IVDR) podría exigir la reevaluación de estas nuevas aplicaciones, especialmente en las enfermedades cardiovasculares.

El papel de la inteligencia artificial en la reutilización de biomarcadores y fármacos: La inteligencia artificial (IA), sumada a las tecnologías de aprendizaje automático, está adquiriendo un papel cada vez más relevante en el campo de la reutilización de fármacos, pudiendo aplicarse estos mismos principios a la reutilización de biomarcadores. Los algoritmos de IA pueden manejar un volumen enorme de datos biomédicos a la hora de identificar posibles biomarcadores aplicables a otras patologías. Esta estrategia ofrece una ventaja significativa en el contexto de las enfermedades raras, sobre las cuales se suele disponer de escasos datos [2]. Por ejemplo, las plataformas de IA han logrado identificar nuevas aplicaciones de fármacos para el tratamiento de enfermedades raras, un proceso que se podría aplicar también a los biomarcadores [2]​. Al analizar patrones en datos de pacientes, incluida la información genética, proteómica y clínica, las herramientas de IA podrían ayudar a identificar nuevas aplicaciones de biomarcadores ya existentes, como el CA125, acelerando el proceso de descubrimiento y transferencia a la práctica clínica.

Implicaciones clínicas y direcciones futuras: El éxito de la reutilización de biomarcadores como el CA125 podría tener profundas implicaciones en la clínica. En la IC, donde resulta crucial contar con biomarcadores precisos para establecer un pronóstico y orientar las decisiones terapéuticas, el CA125 se postula como una nueva herramienta que mejorará la atención al paciente. Su capacidad para reflejar la congestión y la inflamación la convierte en un valioso complemento de otros marcadores ya validados, como el NT-proBNP ​ [8], 10]. La incorporación del CA125 en los protocolos clínicos permitiría a los facultativos estratificar con mayor precisión a sus pacientes en función de sus riesgos y adaptar consecuentemente los tratamientos. Así mismo, el concepto de reutilización de biomarcadores podría ampliarse a otros campos de la medicina. A medida que vamos ahondando en nuestro conocimiento de los mecanismos compartidos por distintas enfermedades, los biomarcadores originariamente asociados a una patología podrían encontrar una nueva vida en otras patologías, creando nuevas oportunidades para el diagnóstico, seguimiento y tratamiento de una amplia variedad de enfermedades. La reutilización de biomarcadores no atañe únicamente al CA125, sino que es aplicable a otros biomarcadores, como la Galectina-3 en la inflamación y la procalcitonina en la sepsis y la oncología. En conclusión, la reutilización de biomarcadores como el CA125 posee un gran potencial en el campo de la medicina personalizada. La reutilización de biomarcadores ya existentes para otros fines permitiría acelerar el desarrollo de nuevas herramientas diagnósticas y terapéuticas, mejorando los resultados clínicos en una gran diversidad de patologías. Con la evolución de la investigación y la integración de la IA, así como de otras tecnologías avanzadas, van a acelerar este proceso, convirtiendo la reutilización de biomarcadores en un elemento clave de la innovación clínica.
